# Whole-Genome Thermodynamic Analysis Reduces siRNA Off-Target Effects

**DOI:** 10.1371/journal.pone.0058326

**Published:** 2013-03-06

**Authors:** Xi Chen, Peng Liu, Hui-Hsien Chou

**Affiliations:** 1 Department of Genetics, Development and Cell Biology, Iowa State University, Ames, Iowa, United States of America; 2 Department of Statistics, Iowa State University, Ames, Iowa, United States of America; 3 Department of Computer Science, Iowa State University, Ames, Iowa, United States of America; Shantou University Medical College, China

## Abstract

Small interfering RNAs (siRNAs) are important tools for knocking down targeted genes, and have been widely applied to biological and biomedical research. To design siRNAs, two important aspects must be considered: the potency in knocking down target genes and the off-target effect on any nontarget genes. Although many studies have produced useful tools to design potent siRNAs, off-target prevention has mostly been delegated to sequence-level alignment tools such as BLAST. We hypothesize that whole-genome thermodynamic analysis can identify potential off-targets with higher precision and help us avoid siRNAs that may have strong off-target effects. To validate this hypothesis, two siRNA sets were designed to target three human genes IDH1, ITPR2 and TRIM28. They were selected from the output of two popular siRNA design tools, siDirect and siDesign. Both siRNA design tools have incorporated sequence-level screening to avoid off-targets, thus their output is believed to be optimal. However, one of the sets we tested has off-target genes predicted by Picky, a whole-genome thermodynamic analysis tool. Picky can identify off-target genes that may hybridize to a siRNA within a user-specified melting temperature range. Our experiments validated that some off-target genes predicted by Picky can indeed be inhibited by siRNAs. Similar experiments were performed using commercially available siRNAs and a few off-target genes were also found to be inhibited as predicted by Picky. In summary, we demonstrate that whole-genome thermodynamic analysis can identify off-target genes that are missed in sequence-level screening. Because Picky prediction is deterministic according to thermodynamics, if a siRNA candidate has no Picky predicted off-targets, it is unlikely to cause off-target effects. Therefore, we recommend including Picky as an additional screening step in siRNA design.

## Introduction

RNA interference (RNAi) is a post-transcriptional gene silencing phenomenon caused by double-stranded RNAs. In mammalian organisms, RNAi is mainly triggered by small interfering RNA (siRNA) or microRNA (miRNA) [1]–[3]. In siRNA duplexes, sense and anti-sense strands are perfectly matched. The anti-sense strand, also named the *guide strand*, is incorporated into a RNA-Induced Silencing Complex (RISC) and activates it, whereas the sense strand, also named the *passenger strand*, is cleaved and destroyed [4]. The anti-sense strand guides the activated RISC to mRNA targets and induces gene silencing [5]. There are many rubrics for high efficient siRNA design. Most importantly, the 5′ anti-sense end should have lower internal thermodynamic stabilities than the 3′ anti-sense end. This assists correct strand incorporation with RISC [6]. In addition, the GC content, the accessibility of target sites and the absence of palindromes or internal repeats should also be considered [7]–[10]. Finally, specific base preferences for RISC incorporation are also important factors for good siRNA design [4], [11].

Although many existing siRNA design software tools have considered all the design issues mentioned above, there is a remaining challenge in siRNA design – the prevention of off-target effects [12]–[14]. A siRNA candidate can be designed to satisfy all rules above, but we still cannot exclude its off-target effects on other unintended genes. There are two categories of off-target effects according to their origins [15]. Off-target effects can be caused by high complementary between siRNA and a nontarget gene – this is named *type I off-target effect*. The other category of off-target effects is caused by the seed region of siRNA – this is named *type II off-target effect*. Seed region is recognized on miRNAs and contains the 2nd to 8th or 9th nucleotides (nt) of a miRNA guide strand [16]. Although the biogenesis of miRNA and siRNA is different, their downstream RNA interference pathways are merged [17]. Therefore, siRNAs can act like miRNAs and down-regulate gene expression through perfect hybridizations in their seed region [17], [18]. So far, no computational method is able to prevent type II off-target effects due to the extremely high false-positive rate. In this paper, we discuss a novel method to prevent type I off-target effect.

There are several existing methods to identify potential off-target genes that may be subjected to type I off-target effects. Most siRNA design software such as siDesign (http://www.dharmacon.com/designcenter/designcenterpage.aspx), siRNA Target Finder [19] and Gene Specific siRNA Selector [20] use BLAST to screen for off-target genes. However, BLAST may overlook some potential off-targets or rank them inconsistently from their off-targeting potential determined by thermodynamics, thus it cannot prevent all off-target silencing effects (see Table S4 and [21], [22]). Alternatively, siDirect calculates the minimum number of mismatches between a siRNA sequence and any nontarget sequence using an improved search algorithm which was said to be more sensitive than BLAST [22], [23]. Nevertheless, the mismatch based off-target screening can still overlook potential off-targets when they have higher mismatches or rank them inconsistently from their off-targeting potential determined by thermodynamics (Table S4). Disregard the differences in time and precision, these off-target search methods all lack thermodynamic consideration. This is a critical shortcoming because molecules bind to each other not because of how many base-pairs matched but because they can form stable duplexes under the thermodynamic conditions including temperature, partner concentrations, and salt effects [24]–[26]. siRNAs are very short oligonucleotides, thus their thermodynamic properties tend to fluctuate [26], [27]. Even at the same sequence similarity level to other genes, some siRNAs can exert strong off-target effects but the others will not have any effect. A 19 base-pair siRNA has been shown to silence an off-target gene with 8 base-pair mismatches [12]. However, we cannot throw out all siRNA candidates that have 8 or less base-pair mismatches to any off-target gene in a large gene set, because that will render most genes without suitable siRNAs to target. Therefore, sequence comparison is not a good indicator of potential off-target effects.

To address this issue, we adopt a whole-genome thermodynamic analysis software Picky for siRNA design and show that it can precisely predict off-targets. Picky efficiently finds unique regions on some interested genes against a complex genome background [27], [28]. The uniqueness is determined by thermodynamics, not by sequence similarity. Therefore, any probe designed to hybridize to these unique regions will only hybridize to the interested genes. Picky employs hundreds of parameters biochemists have empirically determined to conduct its thermodynamic calculations according to the Nearest-Neighbor Model [29]–[32]. In addition to perfect Watson-Crick base-parings, Picky also considers mismatches (e.g., G•A, G•G or G•T) [31], [33]–[36], dangling ends [37], and gap mismatches (i.e., bulge or loop) [38]. This capability makes Picky the microarray design software of choice by many researchers [39]–[42]. Picky also provides an Examine function which can screen a set of predetermined short probe sequences against a large gene set, which is usually a whole genome or transcriptome [43]. Examination using Picky is very efficient. Even for large gene sets like the human’s, it only takes just a few minutes on a modern computer. Output from Picky is a ranked list of target genes with perfect matches to the probes and nontarget genes with imperfect but thermodynamically significant matches to the same probes. The list is sorted in descending order according to the calculated melting temperatures where each target or nontarget gene can hybridize to a probe. Therefore, the Examine function of Picky can be utilized to predict the off-target genes of siRNAs. For example, siRNAs designed by any software can be given as the probe set to Picky and compared against the human transcriptome set. The output for each siRNA will be a list of its target genes and any nontarget genes that are potential off-targets. The difference between the target and nontarget melting temperatures calculated for each nontarget gene is designated its *thermodynamic score*. The lowest thermodynamic score among all nontargets of a siRNA candidate becomes its *siRNA score*. We hypothesize that if a siRNA score is low, it has greater potential to hybridize to its lower scoring off-target genes. Conversely, if a siRNA score is high, it is less likely to hybridize to any off-target. Based on our previous studies, a 10°C difference between target and off-target melting temperatures provides a reasonable safe margin to distinguish good microarray probes [28], [43]. Therefore we used the same threshold in this study. Nevertheless, this threshold can be set to any value preferred by the users.

**Figure 1:**
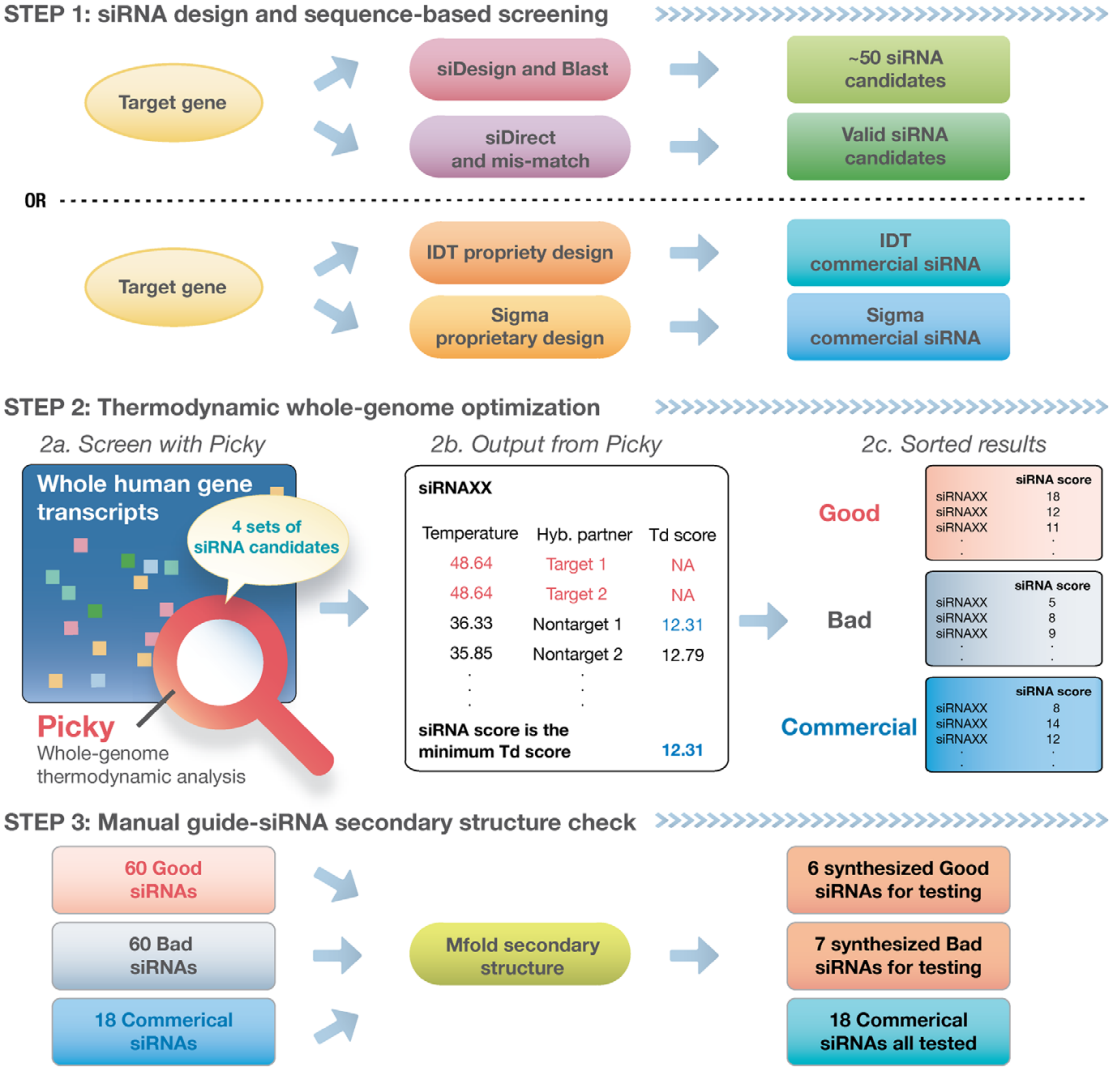
Workflow for designing, checking and selection of testing siRNAs. In STEP 1, siRNA candidates are designed using siDesign and siDirect with their sequence-level screening turned on. Commercial siRNAs are designed by the vendors using their proprietary design pipelines which presumably also include sequence-level screening. In STEP 2, all siRNA candidates are examined by Picky to discover any potential off-target genes. Testing siRNAs are separated into the *good* and *bad* sets according to their siRNA score, i.e., the identified minimum difference between target and nontarget hybridization temperatures. Off-target genes for commercial siRNAs are similarly predicted by Picky. In STEP 3, the anti-sense strand secondary structures of the top 60 good and bad siRNA candidates are checked by the Mfold server, and 6 good and 7 bad siRNAs are selected for the validation experiments. All 18 commercial siRNAs are used in the experiments.

To validate this hypothesis and the use of Picky for siRNA off-target prediction, we have followed the three steps depicted in Fig. 1 to design several siRNAs in our study. In STEP 1, we generated siRNA candidates to target three human genes IDH1, ITPR2 and TRIM28 using two popular online siRNA design tools siDesign (http://www.dharmacon.com/designcenter/designcenterpage.aspx) and siDirect [22]. Note that both siRNA design tools have already incorporated sequence-level screening with BLAST or mismatch calculation to prevent off-target effects. In STEP 2, we used Picky to screen the resulted siRNA candidates and predict any potential off-targets that were missed. Each predicted off-target gene of a siRNA has an associated thermodynamic score and the siRNA itself is assigned a siRNA score. Two distinct siRNA sets were selected according to Picky screening: a *good* siRNA set containing only siRNAs whose scores are higher than 10, and a *bad* siRNA set containing siRNAs whose scores are below 10. In STEP 3, 60 bad siRNAs with the lowest scores and 60 good siRNAs with the highest scores were sent through the Mfold sever to examine their siRNA secondary structures [44]. STEP 3 aims at removing structurally ineffective siRNAs and improving our results. At the end, 6 good and 7 bad siRNAs were selected for subsequent experiments. In addition, we have ordered 18 commercial siRNAs from Integrated DNA Technology (IDT) and Sigma-Aldrich (Sigma) for the same target genes IDH1, ITPR2 and TRIM28. These commercial siRNAs were designed by the vendors and have also been screened for potential off-target effects [45], [46]. They were also put through Picky screening in STEP 2 to identify any potential off-targets that were missed. Their siRNA secondary structures were also examined in STEP3 but all of them were used in subsequent experiments disregard their secondary structures.

The designation of good or bad siRNAs in our selection refers to their predicted off-target effects, not their targeting efficiency. Picky predicted that there are a total of 19 potential off-target genes for the 7 bad siRNAs (Table 1). Among these off-targets, 3 were not tested because their thermodynamic scores are very close to 10, thus they are less likely to be subjected to off-target effects. Another off-target gene has changed annotation since our design, thus it is no longer a viable off-target. In total, we tested 15 off-targets and found 12 to be expressed at detectable levels. Among the 12, our qPCR results indicated that 7 were significantly reduced by the siRNAs.

**Table 1:** Picky predicted siRNA off-target genes.

siRNA name	Potential off-target genes	Thermo-dynamic score	Relative levels of off-targets	Remark	Mismatches reported by siDirect	Ranking in BLAST output
IDH1-B-direct	SYBU	6.22	1.17	Cp = 29	Not reported	
	CEP97	8.49	1.37	Cp = 24	Not reported	
IDH1-B-design-2	OTOG	6.63	1.21	Cp = 29		Not reported
IDH1-B-design-3	**MAPK10**	6.94	0.58	Cp = 29		1
ITPR2-B-Direct	**ITPR1**	5.54	0.87	Cp = 24	2	
	*UHRF1BP1L*	8.65	N/A	Cp>30	1	
	*PLCXD3*	9.76	N/A	N/A	3	
	*DST*	9.92	N/A	N/A	3	
ITPR2-B-Design	*ARHGEF10L*	3.41	N/A	Cp>30		14
	TTYH2	6.61	1.04	Cp = 25		20
	**UBE2R2**	6.85	0.42	Cp = 21		1
	**TPR**	6.95	0.88	Cp = 20		2
	*SFSWAP*	7.89	N/A	Cp>30		170
	**NCAPH**	8.44	0.89	Cp = 21		25
	*CAPN2*	8.96	N/A	N/A		31
	*BNC2*	9.74	N/A	N/A		157
TRIM28-B-design-1	**MMP2**	5.48	0.84	Cp = 24		3
TRIM28-B-	MICAL3	5.90	0.95	Cp = 23		172
design-2	**DNAJC6**	7.40	0.82	Cp = 24		7
	*C17orf85*	9.58	N/A	N/A		Not reported
TRIM28-1 (IDT)	*PDZRN3*	7.96	N/A	Cp>30		
ITPR2-1 (Sigma)	*ZNF622*	9.32	N/A	N/A		
TRIM28-1	EXT2	7.79	1.03	Cp = 20		
(Sigma)	GGA1	8.93	1	Cp = 23		
TRIM28-2	**SOGA1**	2.51	0.89	Cp = 27		
(Sigma)	TSPAN7	7.35	0.9	Cp = 23		
	PDE4DIP	8.27	1	Cp = 26		
	*MED12*	9.11	N/A	N/A		

List of off-target genes predicted by Picky for the bad siRNAs and four commercial siRNAs. Only detectable off-target genes (Cp values smaller than 30) are labelled with their qPCR measured Cp values. Off-target genes whose Cp value is larger than 30 are so indicated and excluded from further consideration. Off-targets with confirmed reductions are shown in **bold font face**.

## Results

### Selection of Testing siRNAs

**Figure 2:**
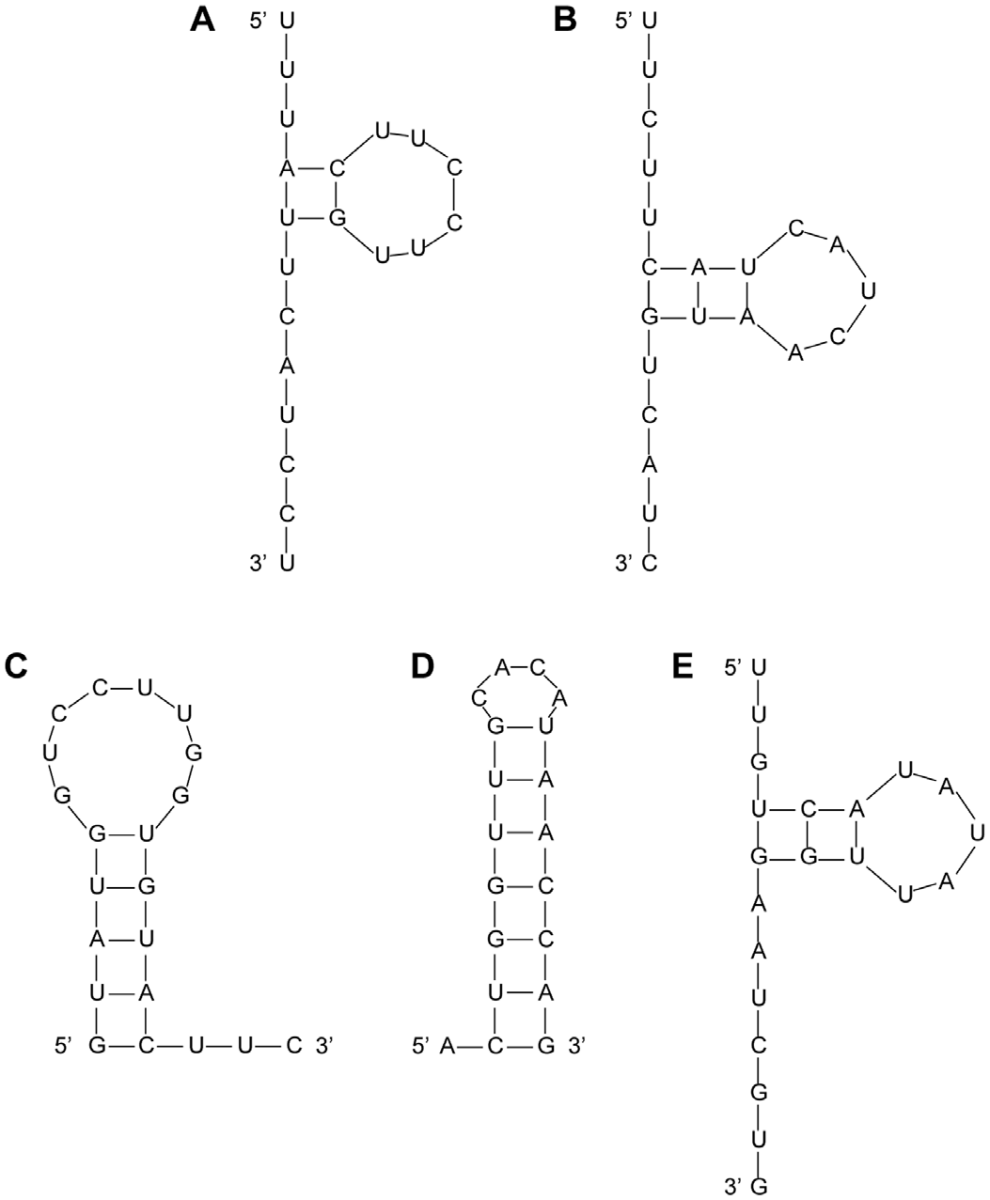
The secondary structures of a few representative siRNAs. Examples of siRNAs with active secondary structures and accessible 5′- and 3′-ends: (A) ITPR2-G-design ΔG = 1.40 kcal/mol and (B) ITPR2-B-design ΔG = 1.60 kcal/mol. Commercial siRNAs with unfavourable secondary structures and thus reduced potency: (C) TRIM28-1 (IDT) ΔG = −0.50 kcal/mol and (D) TRIM28-3 (Sigma) ΔG = −6.60 kcal/mol. An interesting exception of a siRNA with active secondary structure but reduced potency: (E) ITPR2-1 (IDT) ΔG = 0.40 kcal/mol. All secondary structures and the Gibbs free energy (ΔG) values are obtained from the Mfold server.

We have selected three well-annotated genes IDH1, ITPR2 and TRIM28 as the siRNA targets. They all have sufficient RNA expression levels in the HEK293 human cell line. Moreover, each of them has only one transcript isoform, which simplifies siRNA design. Using two popular online software tools siDirect and siDesign, we have obtained siRNA candidates for the three genes. After Picky screening, these siRNA candidates are separated into good and bad sets. Good siRNAs have scores higher than 10, whereas bad siRNAs have scores lower than 10. Previous studies suggested that the secondary structure of siRNA anti-sense strand is also important for the successful hybridization between siRNAs and target sequences [47]. Therefore, the top 20 siRNA candidates from both the good and bad sets for each target gene are screened by Mfold to determine the ones with favorable binding structures [44]. Only siRNA candidates with sufficient length of overhangs at both the 5′ and 3′ ends of the anti-sense strand are considered (c.f., Fig. 2A and 2B). Finally, we select two good siRNAs for each target gene: one from siDirect designed siRNAs and the other from siDesign designed siRNAs (Table S1 and Table S2). Following a three part naming convention (target gene)-(good or bad choice)-(design software), the 6 good siRNAs are named IDH1-G-direct, IDH1-G-design, ITPR2-G-direct, ITPR2-G-design, TRIM28-G-direct and TRIM28-G-design respectively. Here the -direct and -design suffixes refer to the siDirect and siDesign software. These good siRNAs satisfy two notable conditions: their siRNA scores are higher than 10, and their secondary structures favor siRNA efficiency. We also select several bad siRNAs for each target gene, which are named IDH1-B-direct, IDH1-B-design-2, IDH1-B-design-3, ITPR2-B-direct, ITPR2-B-design, TRIM28-B-design-1 and TRIM28-B-design-2 respectively (Table S1). An additional numeral suffix is added if two bad siRNAs would otherwise share the same name. These bad siRNAs also satisfy two conditions: their siRNA scores are below 10, but their secondary structures still favor siRNA efficiency. To summarize, the only difference between good and bad siRNAs is their Picky computed siRNA scores. Otherwise, they are the same quality siRNAs designed by popular tools that satisfy all siRNA design rules. In total, we have synthesized 13 siRNAs corresponding to the three target genes IDH1, ITPR2 and TRIM28 for the following experiments.

### Check the Potential Off-target Genes of siRNAs

**Figure 3:**
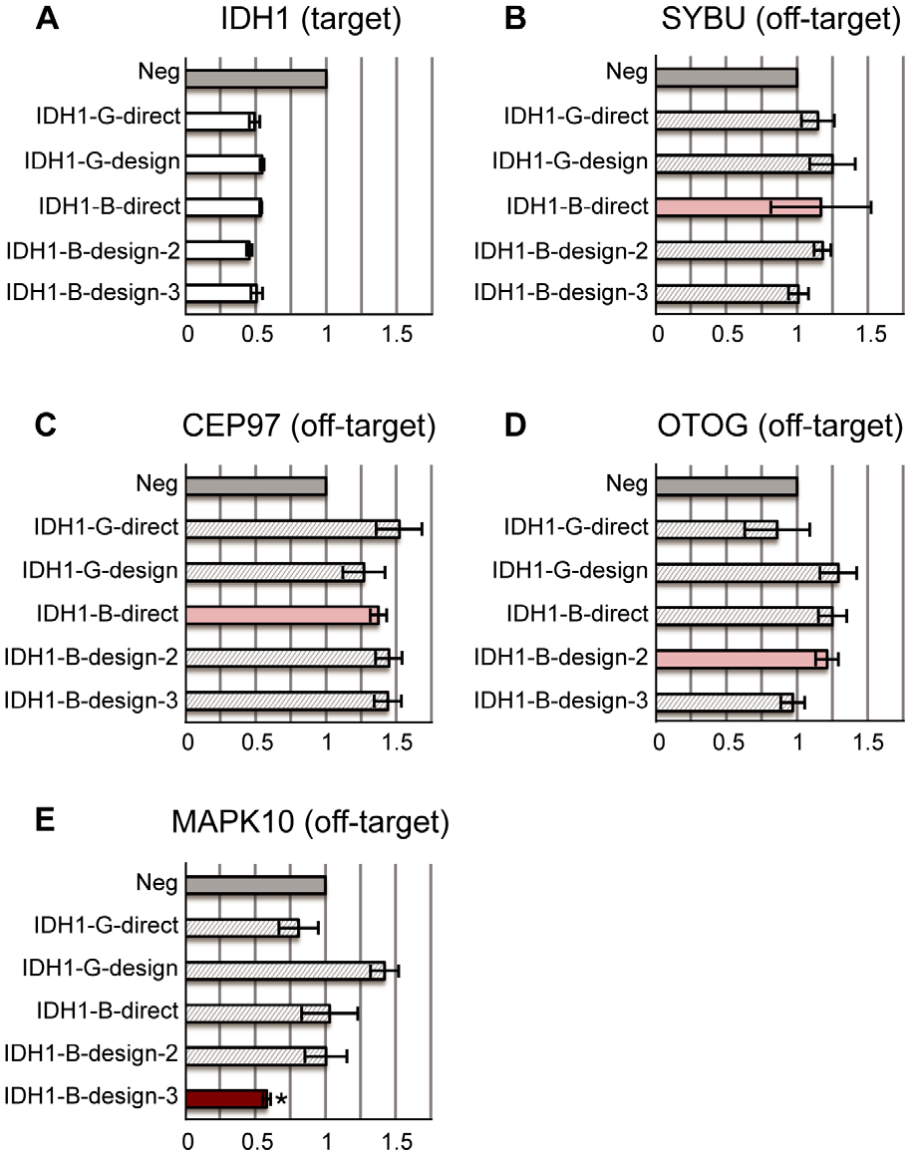
siRNAs targeting IDH1. (A) The relative expression levels of the target gene: IDH1. Two good siRNAs (-G-) and three bad siRNAs (-B-) are designed to target the IDH1 gene; all of them decrease the target gene expression by about 50%. (B–E) The relative expression levels of off-target genes predicted by Picky. Different subfigures correspond to different off-target genes that are associated with different siRNAs respectively. All expression levels are calibrated to the negative transfection controls (Neg: dark grey bars). The light grey bars correspond to treatments with siRNAs except the one with off-target effect predicted by Picky. A test was performed for significant reduction of gene expression by the siRNA with predicted off-target effect compared with other siRNAs. If p-value <0.05, the treatment using siRNA with the predicted off-target effect is red and labelled by *. Otherwise, it is pink. Remarkably, MAPK10, the predicted off-target gene of IDH1-B-design-3, has about 40% reduction in expression level by IDH1-B-deisgn-3.

**Figure 4:**
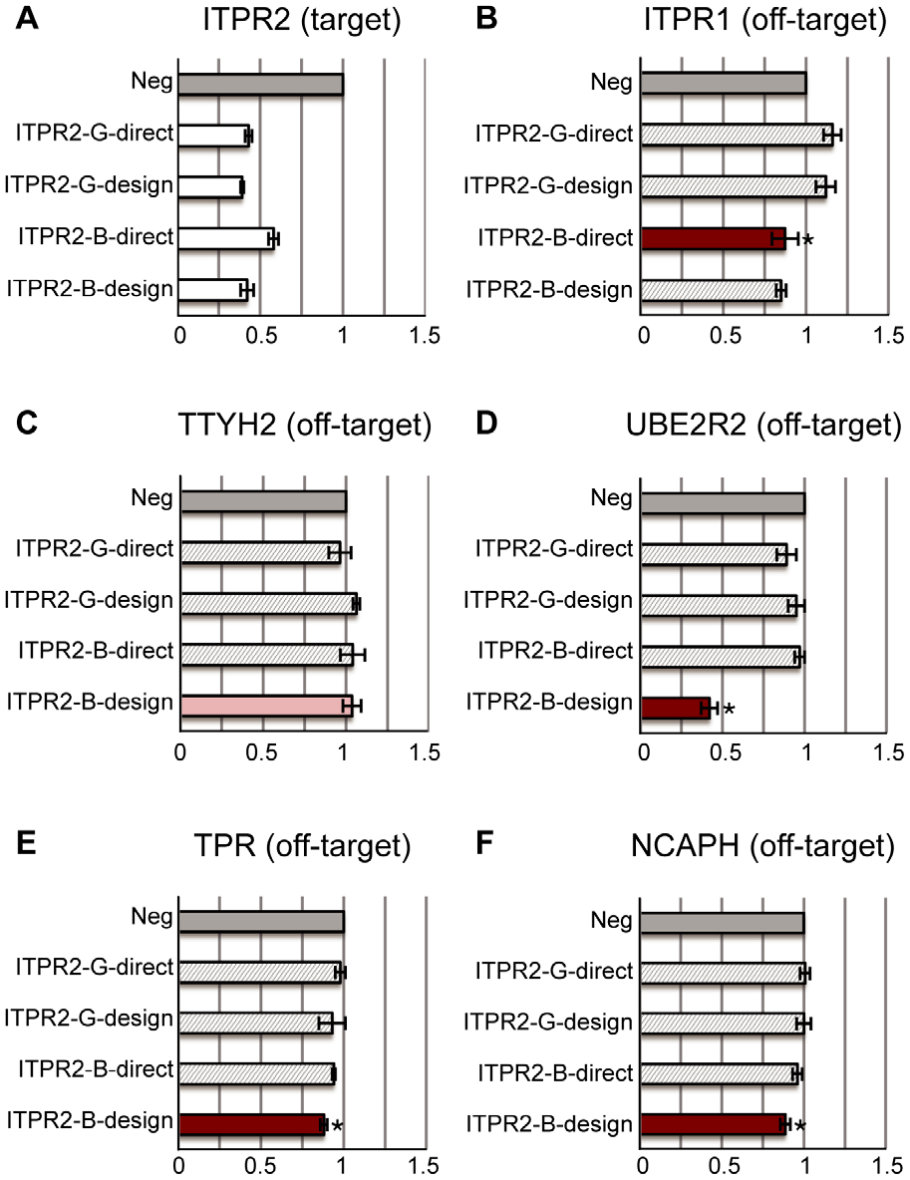
siRNAs targeting ITPR2. (A) The relative expression levels of the target gene: ITPR2. Two good siRNAs (-G-) and two bad siRNAs (-B-) are designed to target the ITPR2 gene. Most of them can reduce the target gene expression level to below 50%. (B–F) The relative expression levels of off-target genes predicted by Picky. Different subfigures correspond to different off-target genes that are associated with different siRNAs respectively. All expression levels are calibrated to the negative transfection controls (Neg: dark grey bars). The light grey bars correspond to treatments with siRNAs except the one with off-target effect predicted by Picky. A test was performed for significant reduction of gene expression by the siRNA with predicted off-target effect compared with other siRNAs. If p-value <0.05, the treatment using siRNA with the predicted off-target effect is red and labelled by *. Otherwise, it is pink. Remarkably, the expression level of UBE2R2 was reduced by 60% by ITPR2-B-design.

Our qPCR results indicate that most siRNAs, whether good or bad ones, work efficiently in silencing their target genes. All good siRNAs decrease target gene expressions to around 50% (Fig. 3A, 4A, and 5A). Similarly, all bad siRNAs reduce target gene expressions efficiently to around 50% (Fig. 3A, 4A, and 5A). These results confirm that all selected siRNAs are functional and there is no bias in their selections.

**Figure 5:**
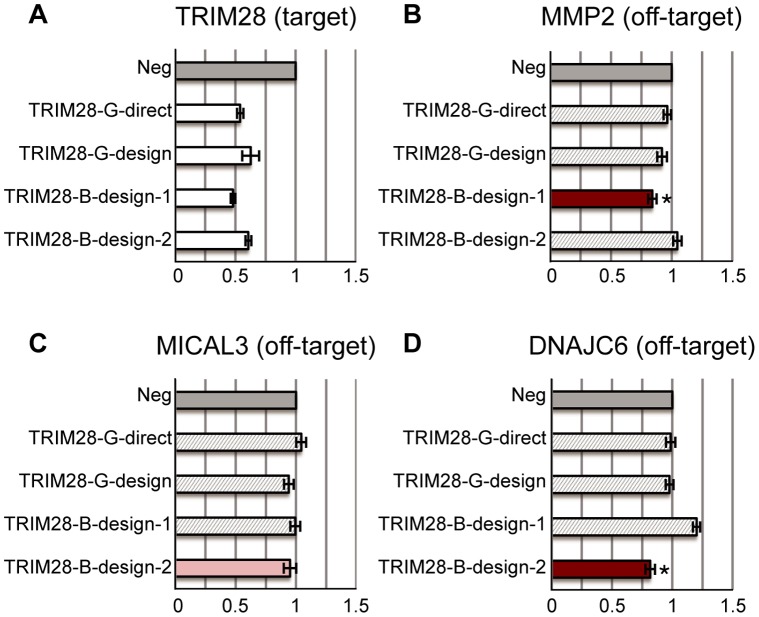
siRNAs targeting TRIM28. (A) The relative expression levels of the target gene: TRIM28. Two good siRNAs (-G-) and two bad siRNAs (-B-) are designed to target the TRIM28 gene. All of them reduce the target gene expression level. (B–D) The relative expression levels of off-target genes predicted by Picky. Different subfigures correspond to different off-target genes that are associated with different siRNAs respectively. All expression levels are calibrated to the negative transfection controls (Neg: dark grey bars). The light grey bars correspond to treatments with siRNAs except the one with off-target effect predicted by Picky. A test was performed for significant reduction of gene expression by the siRNA with predicted off-target effect compared with other siRNAs. If p-value <0.05, the treatment using siRNA with the predicted off-target effect is red and labelled by *. Otherwise, it is pink.

Picky predicts that IDH1-B-design-3 may have an off-target gene MAPK10 (Table 1). qPCR shows that MAPK10 expression is indeed decreased significantly in IDH1-B-design-3 treated cells. This reduction is not likely caused by IDH1 pathway effects because cells treated by the other three siRNAs targeting IDH1 do not exhibit reduced MAPK10 levels (Fig. 3E). The other predicted off-target genes SYBU, CEP97 and OTOG remain stable under the treatments of IDH1-B-direct and IDH1-B-design-2 (Table 1 and Fig. 3B–D). Some off-target gene expressions are increased in IDH1 experiments. It was suggested that IDH1 is a member of oncogenic signalling pathways involved in gliomagenesis [48]. Therefore, the down-regulation of IDH1 may cause distortion of normal metabolism processes, leading to abnormal expressions of some genes.

ITPR2-B-direct has three predicted off-target genes ITPR1, UHRF1BP1L, PLCXD3 and DST (Table 1). qPCR shows the amplification crossing point (Cp) values of UHRF1BP1L are beyond 30. We cannot obtain reliable results at high Cp values (i.e., low gene expressions). Therefore, if the average Cp value of a gene is over 30, we do not consider it further. PLCXD3 and DST are not considered either because their thermodynamic scores are close to the good siRNA cutoff score 10, making them a borderline off-target. ITPR1 expression in ITPR2-B-direct treated cells is decreased by 30% when compared to the other two siRNAs ITPR2-G-direct and ITPR2-G-design (Fig. 4B), which actually increase ITPR1 expression to 116% and 112%. ITPR1 and ITPR2 both participate in inositol 1, 4, 5-triphosphate signalling pathway. When ITPR2 expression is decreased in siRNA treated cells, ITPR1 is up-regulated to compensate [49]. ITPR1 expression is expected to increase in ITPR2-G-direct and ITPR2-G-design treated cells, but it is still decreased in ITPR2-B-direct treated cells due to off-target effect as predicted. Interestingly, ITPR1 expression is also decreased in ITPR2-B-design treated cells, but it is not the predicted off-target for ITPR2-B-design. Further analysis suggests that this may be caused by the off-target effect from the seed region of ITPR2-B-design (Fig. S1).

ITPR2-B-design has several predicted off-target genes ARHGEF10L, TTYH2, UBE2R2, TPR, SFSWAP, NCAPH, CAPN2 and BNC2 (Table 1). ARHGEF10L and SFSWAP are not further considered due to their high Cp values. CAPN2 was predicted to be an off-target when GRCh37.64 human transcript data set was used. In the updated GRCh37.66 data set the annotation of CAPN2 has changed, thus CAPN2 is no longer a predicted off-target gene. BNC2 is not considered either because its thermodynamic score 9.74 is very close to the good siRNA cutoff score 10. We only report the qPCR results with the remaining 4 off-target genes TTYH2, UBE2R2, TPR and NCAPH. The latter three expression levels are all decreased to various levels in ITPR2-B-design treated cells (Fig. 4D–F) – UBE2R2 has an extremely strong 60% reduction close to the reduction of the intended target gene ITPR2. Only TTYH2 expression does not significantly change in ITPR2-B-design treated cells (Fig. 4C).

The last target gene TRIM28 has two bad siRNAs. TRIM28-B-design-1 has one predicted off-target gene MMP2, and TRIM28-B-design-2 has three predicted off-target genes MICAL3, DNAJC6 and C17orf85 (Table 1). The thermodynamic score of C17or85 is 9.58 thus it is excluded. qPCR results indicate that MMP2 expression is reduced significantly in TRIM28-B-design treated cells when compared to the other three siRNAs (Fig. 5B). DNAJC6 is decreased in TRIM28-B-design-2 treated cells (Fig. 5D), but MICAL3 expression level did not differ noticeably (Fig. 5C).

### Check the Potential Off-target Genes of Commercial siRNAs

To test if our method also works on preventing off-targets for commercial siRNAs, some siRNAs for the same target genes IDH1, ITPR2 and TRIM28 were purchased from Sigma (MISSION siRNA) and IDT (TriFECTa Kit). For each target gene, the top three recommended siRNAs from Sigma and IDT were ordered. Sigma siRNAs are traditional siRNA duplexes 19 nt in length with two dTdT overhangs at the 3′ end of both strands. Only the 19 nt RNA part is screened by Picky. IDT siRNAs have a special design for Dicer cutting which may enhance their potency [45]. IDT siRNA sense strand is 25 nt and anti-sense strand is 27 nt with 2 nt overhang at the 3′ end. We chose the 21 nt sequence after Dicer cutting as the sequence to be screened by Picky.

**Figure 6:**
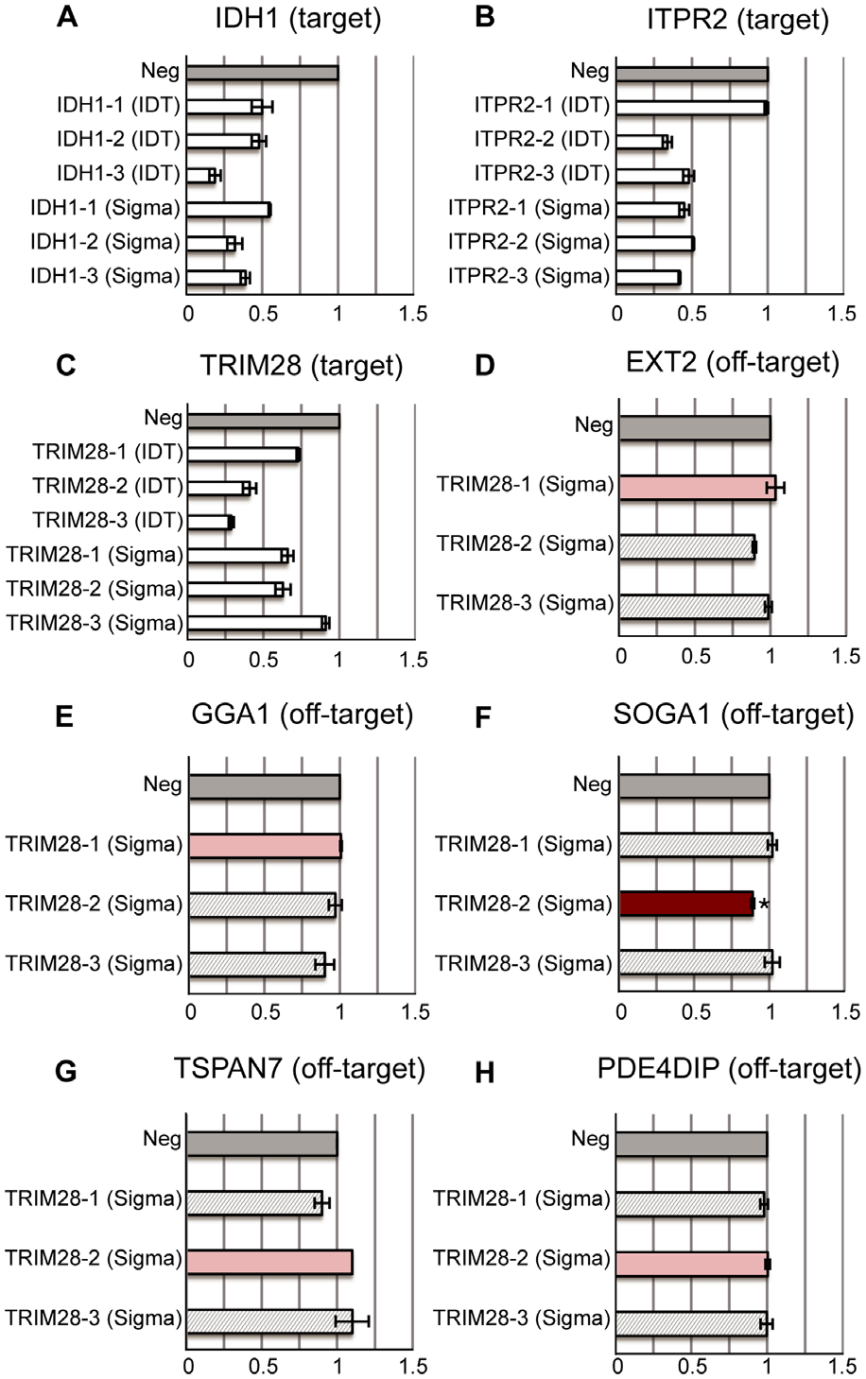
Commercial siRNAs. (A–C) 18 commercial siRNAs are purchased from Sigma (MISSION siRNA) and IDT (TriFECTa Kit) to target the same genes IDH1, ITPR2 and TRIM28. The commercial siRNA potency on target genes is mostly comparable to that of our siRNAs. (D–H) The relative expression levels of predicted off-target genes in different Sigma siRNA treated cells (IDT siRNAs are not included in off-target analyses). All expression levels are calibrated to the negative transfection controls (Neg: dark grey bars). The light grey bars correspond to treatments with siRNAs except the one with off-target effect predicted by Picky. A test was performed for significant reduction of gene expression by the siRNA with predicted off-target effect compared with other siRNAs. If p-value <0.05, the treatment using siRNA with the predicted off-target effect is red and labelled by *. Otherwise, it is pink. The SOGA1 off-target gene is significantly reduced by TRIM28-2 (Sigma).

Most commercial siRNAs for IDH1 and ITPR2 can reduce their target gene level to 50% or less (Fig. 6A–B). TRIM28 siRNAs are relatively weak (Fig. 6C). In total, three commercial siRNAs ITPR2-1 (IDT), TRIM28-1 (IDT) and TRIM28-3 (Sigma) cannot efficiently reduce target gene expressions. TRIM28-1 (IDT) and TRIM28-3 (Sigma) are forming stem-loops without enough overhangs at the 5′ and 3′ ends according to Mfold (Fig. 2C and 2D). In particular, the Gibbs free energy of the secondary structure of TRIM28-3 (Sigma) is −6.6 kcal/mol, which is too strong to unwind. The secondary structure of ITPR2-1 (IDT) satisfies the RISC-favorable secondary structure rules (Fig. 2E) but it is still low efficient. A possible explanation is that the targeting site on ITPR2 is not accessible by ITPR2-1 (IDT) [50].

We used Picky to predict the potential off-target genes of commercial siRNAs. Only one IDT siRNA TRIM28-1 (IDT) has a predicted off-target gene PDZRN3. Because PDZRN3 has a high Cp value, we do not consider it further. Only three Sigma siRNAs ITPR2-1 (Sigma), TRIM28-1 (Sigma) and TRIM28-2 (Sigma) have predicted off-targets. ITPR2-1 (Sigma) has one off-target gene ZNF622 but its thermodynamic score is near 10, thus it is not considered. TRIM28-1 (Sigma) has off-targets EXT2 and GGA1. qPCR indicates both EXT2 and GGA1 expression levels are not reduced in TRIM28-1 (Sigma) treated cells (Fig. 6D and 6E). TRIM28-2 (Sigma) has off-target genes SOGA1, TSPAN7, PDE4DIP and MED12. The thermodynamic score of MED12 is near 10, thus it is not considered. SOGA1 is significantly reduced in TRIM28-2 (Sigma) treated cells (Fig. 6F). TSPAN7 and PDE4DIP expression levels do not change in TRIM28-2 treated cells (Fig. 6G and 6H).

Surprisingly, EXT2 expression is reduced by TRIM28-2 (Sigma) and TSPAN7 expression is reduced by TRIM28-1 (Sigma) (Fig. 6D and 6G). Neither gene is the predicted off-target of the corresponding siRNA. A possible explanation is that the unexpected reduction may be caused by the siRNA seed regions as seen in the hybridizations of these two off-target genes with TRIM28-2 (Sigma) and TRIM28-1 (Sigma) respectively (Fig. S1, Section B).

## Discussion

How to reduce siRNA off-target effect is an important question. BLAST is the most widely used tool to address this issue. However, BLAST and other sequence-level search tools may overlook some off-targets because sequence alignments cannot reflect true off-target silencing potential [21], [22]. The hybridization likelihood between siRNA candidates and all target and nontarget genes should also be evaluated using thermodynamics. Although several siRNA design tools have employed thermodynamics [51], [52], the computational overhead required in thermodynamic calculation prevents most tools from comparing a siRNA candidate with all potential hybridization sites on all genes.

Picky is a microarray design and analysis tool previously developed for large and complex genomes. Picky employs thermodynamics in all its computation and can efficiently identify all unique regions on interested genes against a large genome background. The uniqueness is determined by the hybridization melting temperature difference: the greater the difference between the probe-to-target melting temperature and the highest probe-to-nontarget melting temperature, the more unique a probe targeted region will be. All potential hybridization sites on all genes are compared to obtain the highest nontarget melting temperature of any probe candidate. Picky calculation is deterministic according to thermodynamic equations and there is no statistical modelling in Picky that may introduce stochastic outcomes. Despite the exhaustive search and comparison, Picky is very efficient. Picky screening requires only a couple minutes even for the whole human gene set. The technical details of how Picky conducts its efficient thermodynamic calculation and the experimental validation of Picky predictions have been reported previously [27], [28], [43].

Carrying on this capability, Picky should be able to screen potential off-target genes for siRNA candidates as well. The question is how precise this screening can be in the context of *in vivo* RNA interference. Picky calculation requires several parameters such as the salt and RNA concentrations, which cannot be precisely estimated for living cells. Therefore, Picky estimated melting temperatures likely deviate from true values. One might even ask the fundamental question why melting temperatures can be useful for RNAi experiments that are executed at room temperature far below any estimated target melting temperature. The key answer to the question is that we are using melting temperature estimate as a measurement of molecular hybridization tendency – the higher an estimated melting temperature between two molecules, the more easily they can hybridize to each other. Estimated melting temperatures are never used alone but are always compared to the other estimated melting temperatures to produce meaningful off-target predictions. In another word, although estimated melting temperatures can deviate from true values, their *relative differences* are preserved in Picky calculation.

Previously we have calibrated Picky designed microarrays and found that even at a 10,000∶1 molar concentration ratio between predicted nontargets and targets, Picky designed probes still will not hybridize to the abundant nontarget transcripts if there are sufficient melting temperature difference between them [28], [43]. Therefore, according to our experience, if the target and nontarget melting temperature difference of a siRNA candidate (i.e., its siRNA score) is higher than 10°C, we believe its off-target effect will be minimal. If a siRNA candidate has a melting temperature difference with a nontarget that is significantly lower than 10°C, it may cause off-target effect on the nontarget gene, and it is probably a *bad* siRNA candidate.

To validate this hypothesis, we have designed experiments to specifically look for predicted off-target gene inhibition by the bad siRNAs. Indeed, more than half of the Picky predicted off-target genes exhibit reduced expression levels (Fig. 3, 4, 5). It is suggested that miRNA seed regions matching perfectly to target genes can help modulate the structure of RISC for inhibition [53], [54]. In our experiments, all predicted off-target genes with a perfect match in the seed region are reduced significantly by the bad siRNAs, except SYBU (Fig. S1). We observe that base position 10 of IDH1-B-direct, the siRNA predicted to cause off-target effect on SYBU, is not complementary with the corresponding base on SYBU. Because canonical siRNA-mediated Ago 2 cleavage is at position 10 or 11, the imperfect complementary on this base may block SYBU knockdown by IDH1-B-direct [55], [56]. We have conducted similar predictions on commercially available siRNAs from IDT and Sigma. Because commercial siRNAs must be purchased before we can learn about their sequences, we cannot preselect good or bad commercial siRNAs. As such, most of the 18 commercial siRNAs we have obtained are good siRNAs. Nevertheless, Picky is still able to predict a few off-target genes for them and at least one of the predicted off-target genes exhibits noticeable off-target effect (Fig. 6).

In our experiments, most siRNAs reduce target gene expressions to the 50% level. Although not considered the most potent siRNAs, they serve our validation purpose well – if we can validate predicted off-targets for weaker siRNAs, we can predict and prevent off-targets for stronger siRNAs. Some predicted off-target genes are reduced only 10–30%, but this relatively low reduction still may affect gene network and cell behaviour, and render some RNAi experiments unreliable. For the clinical use of siRNAs, all such unintended reductions must be avoided.

This work demonstrates that some off-target genes predicted by Picky are reduced by bad siRNAs. This suggests that bad siRNAs should not be used in practice. An added benefit of the off-target prediction is that scientists can now have a small subset of genes to monitor and see if off-target effect has happened. This knowledge can help scientists set the proper dosage of siRNAs in biomedical applications to reduce side-effects. siRNAs that have no Picky predicted off-targets should be safer to use, but proving they indeed have no off-targets is difficult. Most whole transcriptome analysis techniques are not sensitive enough to detect the 10–30% off-target expression variations we have observed.

We have shown that using Picky off-target screening in association with existing siRNA design software can reduce potential off-target effects without sacrificing the potency of the siRNAs. Our method is independent of the specific siRNA design software users prefer to use. All Picky versions are free to academic users, and 32-bit Picky versions are also free to commercial users. A modern multi-core desktop computer with sufficient memory will work for most gene sets. We recommend the Picky screening step added to any siRNA design.

## Materials and Methods

### siRNA Procurement

siRNAs designed using siDesign are set to have a GC content range between 30–64% with BLAST screening for off-targets. Customarily, a dTdT protective overhang is added to both siRNA strands to prevent degradation. Because we need to precisely control the hybridization between siRNAs and transcripts, we have added the genuine overhang from the targeted transcript instead. The anti-sense strand of siRNAs is used for Picky screening.

siRNAs designed using siDirect are set to use the Ui-Tei siRNA selection rule, the seed-duplex stability of Max Tm 21.5°C, the human database screening for off-targets, the GC content range between 30–64% (the same as siDesign), and the avoidance of contiguous A’s, T’s, C’s and G’s. Only siRNA candidates meeting all criteria above are output by siDirect. Because siDirect siRNAs come with the two-base overhang, they are screened by Picky without any modification.

All testing siRNAs are synthesized by Sigma. All commercial siRNAs are purchased from Sigma (MISSION siRNA) and IDT (TriFECTa Kit).

### Picky Screening

All siRNAs are screened using the 64-bit version of Picky 2.20 (http://www.complex.iastate.edu/download/Picky/index.html). The human cDNA sequence file (Homo_sapiens.GRch37.64.cdna.all.fa) is downloaded from NCBI and given as the input gene set to Picky. Input gene sequences are properly reverse-complemented within Picky to match the siRNA anti-sense strand direction. The Examine function of Picky is used to identify potential off-target genes for each siRNA candidate. Table 2 lists the parameters set for the Examine function; detail information about each parameter is provided in the Picky dialog that accepts these parameters.

**Table 2:** Picky off-target gene screening parameters.

Maximum oligo size	22	
Minimum oligo size	19	
Maximum match length	15	
Minimum match length	7	Important for nontarget match
Minimum trigger similarity	66	Important for nontarget match
Minimum temp difference	0	
DNA concentration (nanoM)	0.001	
Salt concentration (milliM)	130	Calculated from cell medium
Screen only forward strands	Yes	
se better salt effect equation	Yes	

### Cell Culture and siRNA Transfection

The human embryonic kidney cell line HEK293 is purchased from the American Type Culture Collection (CRL-1573TM) and cultured in Eagle’s Minimum Essential Medium (Sigma, M0643) with supplementary 2 mM L-glutamine penicillin-streptomycin (Invitrogen 10378-016) and 10% FBS (Sigma, F6178). HEK293 cells are transfected in twelve-well plates using Lipofectamine 2000 (Invitrogen). The concentration of all siRNAs used in transfections is 30 nM. Three biological replicates are performed for each siRNA. Transfection efficiency for siRNAs is about 90% as calculated using the positive florescent control GAPDH siRNA (Ambion, AM4650). Two negative control siRNAs (Sigma, SIC001 and IDT, NC1) were used.

### Isolation of RNA and Reverse Transcription

Total RNA is isolated from cells 48 h following transfection (Qiagen RNeasy kit) and quantified using NanoDrop 1000. First-strand cDNA is synthesized from 1 µg of total RNA for 60 min at 50°C using the Superscript III Reverse transcription system (Invitrogen) with Oligo dT primer (Invitrogen).

### Real-time Quantitative PCR

Real-time quantitative PCR (qPCR) is performed with SYBR Green I Master Mix (Roche) using LightCycler 480 II (Roche). All qPCR primer sequences are summarized in Table S3. Human genes usually have multiple alternative splicing forms, and siRNAs may either hybridize to common areas shared by all splicing forms or only to a few unique areas belonging to just one or a few splicing forms. For example, the SOGA1 gene has two alternative splicing forms SOGA1-1 and SOGA1-2, and Picky predicted only SOGA1-1 (NM_080627.2) is off-targeted by TRIM28-2 (Sigma). Therefore, when designing qPCR primers for SOGA1 expression analysis, we only choose the primers from the unique region of SOGA1-1 when compared to SOGA1-2. All siRNAs are aligned with their potential off-target genes before primers are selected for the off-target genes (Fig. S1). The reference genes POLR2A, TBP and RPLP0 are used to normalize the cDNA concentrations of each sample [57]. Two to three technical replicates on qPCR plates are performed for each biological replicate.

The *E*-method relative quantification is used for the data analysis. *E*-method provides more precisely normalized relative quantification of interested gene expressions between control and treated samples. *E*-method takes into consideration the PCR efficiency differences among interested genes and reference genes in its formula [58]:
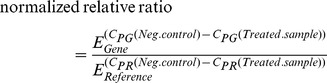



*E_Gene_* and *E_Reference_* are the efficiency values calculated from qPCR standard curves for the interested and reference genes. *C_PG_ (Neg.control)* and *C_PG_ (Treated.sample)* are the averaged raw Cp values from the technical replicates for the interested gene. *C_PR_ (Neg.control)* and *C_PR_ (Treated.sample)* are the averaged raw Cp values from the technical replicates for the reference gene.

### Statistical Analysis

Three biological replicates for each siRNA treatment are performed and quantified by qPCR. A one-sided t-test was performed to test for the difference in the expression level of the off-target gene between treatment using the siRNA associated with the predicted off-target and the treatments using other siRNAs for the same target gene.

## Supporting Information

Figure S1 **Sequence alignments and hybridizations between siRNAs and off-target genes.** FASTA is used for sequence alignments between siRNA sense strand and mRNA for primer design. RNAhybrid is used to identify potential hybridizations between siRNA anti-sense strand seed region and mRNA [59].
(DOCX)

Table S1 **List of all siRNA sequences used in this paper.** Included in the list are the siRNA sources, target genes, ID (if available), names, and sense and anti-sense strand sequences.
(XLSX)

Table S2 **Formatted Picky output for all siRNAs used in this paper.** Picky output is separated into three worksheets: self-designed siRNAs, commercial siRNAs (IDT) and commercial siRNAs (Sigma). Among the listed siRNAs, 11 have predicted off-target genes with thermodynamic scores below 10, and all such off-targets are listed. The other 20 siRNAs have no predicted off-target genes with thermodynamic scores below 10, thus only the off-target gene with the lowest thermodynamic score is listed. The specific meaning of each value in the file is explained at the beginning of the ‘self-design siRNAs’ spreadsheet.
(XLSX)

Table S3 **qPCR primer list.** The specific transcripts that can be amplified by the primers are listed.
(XLSX)

Table S4 **Picky, Mismatch and BLAST off-target screening comparison.** The thermodynamically ranked Picky off-targets for each siRNA candidate are compared to their ranking based on mismatch count (the method adopted by siDirect; see spreadsheet “Mismatch comparison”) and to their ranking based on BLAST (the method adopted by siDesign; see spreadsheet “BLAST comparison”). For mismatch count based off-target prediction, it can be observed that a higher mismatch count does not always reflect the off-targeting tendency predicted by thermodynamics – either a stronger potential off-target can be overlooked because of a higher mismatch count (orange rows), or a weaker potential off-target can be overestimated, thus causing a good siRNA to be rejected, due to a lower mismatch count (the blue row). Generally, mismatch counts do not directly reflect off-targeting potentials (yellow rows). For BLAST based off-target prediction, the small variations in E-values do not reflect the large variations of off-target potential predicted by thermodynamics (orange rows). Some potential off-targets were missed by BLAST even at relaxed search parameters (pink rows). Generally, BLAST ranking does not reflect the thermodynamically predicted off-target potential (yellow or cyan rows).
(XLS)
