# Supplementary material for: Whole-Genome Thermodynamic Analysis Reduces siRNA Off-Target Effects
Source: PLoS One. 2013 Mar 6;8(3):e58326. doi: 10.1371/journal.pone.0058326 (PMC3590146; doi:10.1371/journal.pone.0058326)
Supplement: Figure S1 — Sequence alignments and hybridizations between siRNAs and off-target genes. FASTA is used for sequence alignments between siRNA sense strand and mRNA for primer design. RNAhybrid is used to identify potential hybridizations between siRNA anti-sense strand seed region and mRNA [59]. (DOCX) [file pone.0058326.s001.docx]

### Predicted type I off-target gene *alignments* between siRNA sense strands and mRNA sequences

**SYBU (fourteen alternative isoforms)**

**Sequence alignment between IDH1-B-direct and one of fourteen isoforms of SYBU, NM_001099756.1**

**IDH1-B-direct ATGTGATTTCTCAGAAATTGA**

**X:::::::::: ::::::::X**

**GACTACTCTGTGAAGACTCAATACTCAGAAATGTGATTTCTTAGAAATTGATCCTTGAAG**

**CEP97 (one isoform)**

**IDH1-B-direct ATGTGATTTCTCAGAAATTGA**

**X::::::::::::::X**

**GGTGCCTAAACCTCAGAGTCCTAGATGGATATGTGATTTCTCAGAAGGAAAGTTTGAAAG**

**OTOG (one isoform)**

**IDH1-B-design-2 GAGGGTTAGCCCACAGAGCAA**

**X::::: ::::::::::X**

**TCTCTGCCCACTTCCATGTATGGTTCTGCAGAGGGTGGGCCCACAGAGCTCACGCCTGCT**

**MAPK10 (four alternative splicing forms)**

**Sequence alignment between IDH1-B-design-3 and four splicing forms of MAPK10, NM_138981.2, NM_002753.3, NM_138980.2 and NM_138982.2**

**IDH1-B-design-3 GGAGATATATGACAAGCAGTA**

**X::::::::::::::::X**

**ACCCAGCCGAAGTGGAGGCGCCTCCACCTCAGATATATGACAAGCAGTTGGATGAAAGA**

**ITPR1 (three alternative splicing forms)**

**Sequence alignment between ITPR2-B-direct and three splicing forms of ITPR1, NM_001099952.2, NM_001168272.1 and NM_002222.5**

**ITPR2-B-direct GACAGAACAAAGGAAGAATAA**

**X::::::::::::::X : ::**

**TGGCCAGCTGTCGGAATTAAAGGATCAGATGACAGAACAAAGGAAGCAGAAACAAAGAAT**

**TTYH2 (two alternative splicing forms)**

**Sequence alignment between ITPR2-B-design and two splicing forms of TTYH2, NM_032646.5 and NM_052869.1**

**ITPR2-B-design GATGACATTGATGATGAAGAA**

**X:::::::::::::::X ::**

**AAGCACTTCACCACCAGAAACAGAGACTACGATGACATTGATGATGATGACCCCTTTAAC**

**UBE2R2 (one isoform)**

**ITPR2-B-design GATGACATTGATGATGAAGAA**

**:: X:::::::::::::::X**

**TCAGATTTGCTTTACGACGACTTGTATGATGACGACATTGATGATGAAGATGAGGAGGAG**

**TPR (one isoform)**

**ITPR2-B-design GATGACATTGATGATGAAGAA**

**::::: X:::::::::::X**

**TCAAGGCAAAGGAGATGATGTCATTGTAATTGACAGTGATGATGAAGAAGAGGATGATGA**

**NCAPH (one isoform)**

**ITPR2-B-design GATGACATTGATGATGAAGAA**

**: ::::: X::::::::::X**

**ACCTCCAACTTTTGCCCTGGATTACAGGCTGCTGACAGTGATGATGAAGATTTGGATGAC**

**MMP2 (two alternative splicing forms)**

**Sequence alignment between TRIM28-B-design-1 and two splicing forms of MMP2, NM_001127891.1 and NM_004530.4**

**TRIM28-B-design-1 CCACTGAGGACTACAACCTTA**

**X::::::::::::X :::**

**GCCGCACGGATGGCTACCGCTGGTGCGGCACCACTGAGGACTACGACCGCGACAAGAAGT**

**MICAL3 (three alternative splicing forms)**

**Sequence alignment between TRIM28-B-design-2 and one of three splicing forms of MICAL3, NM_015241.2**

**TRIM28-B-design-2 CATGGAGGTGCAGGAAGGCTA**

**X:::::::::X :: :::::**

**CAGTCATGCCCACCCCAGCATCACAGGAGACATGGAGGTGCGGGCAGGCTCCTGAATTAT**

**DNAJC6 (three alternative splicing forms)**

**Sequence alignment between TRIM28-B-design-2 and three splicing forms of DNAJC6, NM_001256864.1, NM_001256865.1 and NM_014787.3**

**TRIM28-B-design-2 CATGGAGGTGCAGGAAGGCTA**

**: X:::::::::X :::::**

**GCTATGGGGGAGGTCTCTTTGACATGGTAAAAGGAGGTGCAGGGAGGCTCTTTAGTAACC**

**EXT2 (three splicing forms)**

**Sequence alignment between TRIM28-1(Sigma) and three splicing forms of EXT2, NM_000401.3, NM_001178083.1 and NM_207122.1**

**TRIM28-1(Sigma) GCTCTACTGGGCCAGCCAA**

**:::: X:::::::X ::**

**GAGGCTACTTTCTGTGTGGTTCTTCGTGGAGCTCGGCTGGGCCAGGCAGTATTGAGCGAT**

**GGA1 (five splicing forms)**

**Sequence alignment between TRIM28-1(Sigma) and five splicing forms of GGA1, NM_001001560.2, NM_001001561.2, NM_001172687.1, NM_001172688.1 and NM_013365.4**

**TRIM28-1(Sigma) GCTCTACTGGGCCAGCCAA**

**:::: X:::::::::X**

**TGATAGAGCCACGAACCCCCTGAACAAGGAGCTCGACTGGGCCAGCATCAACGGCTTCTG**

**SOGA1 (two alternative splicing forms)**

**Sequence alignment between TRIM28-2(Sigma) and one of two splicing forms of SOGA1, NM_080627.2**

**TRIM28-2(Sigma) GAGACCAAACCTGTGCTTA**

**X:::::::::::X ::::**

**ACTTCCCAGGGGAAGGCAGTGAGTGGGAGAGAGACCAAACCTGGGCTTCCCAAGCATCCA**

**TSPAN7 (one isoform)**

**TRIM28-2(Sigma) GAGACCAAACCTGTGCTTA**

**X:::::::::::::X : : AGTATGGCATCGAGGAGAATGGAGACCAAACCTGTGATAACCTGTCTCAAAACCCTCCTC**

**PDE4DIP (nine splicing forms)**

**Sequence alignment between TRIM28-2(Sigma) and three of nine splicing forms of PDE4DIP, NM_001198832.1, NM_014644.4 and NM_001198834.2**

**TRIM28-2(Sigma) GAGACCAAACCTGTGCTTA**

**::: X::::::::::X :**

**GCCAGACCAACTCTGTTGAAATTCTTGCATAGAGCAAACCTGTGCTCATTTTTAAGTGGC**

### Potential type II off-target *hybridizations* between siRNA anti-sense strand seed regions and mRNA sequences

**OTOG (one isoform)**

**20 10**

**IDH1-B-design-3 3’CCUCUAUAUACUGUUCGUCAU5’**

**|||||||||||**

**5’CCGGAAAUCCUAGUCCAGAGAGCUUCCUGGAUGACAAGCAGGAGGUCCACACAUGGCGAG3’**

**ITPR1 (three alternative splicing forms)**

**Sequence alignment between ITPR2-B-design and three splicing forms of ITPR1, NM_001099952.2, NM_001168272.1 and NM_002222.5**

**20 10**

**ITPR2-B-design 3’CUACUGUAACUACUACUUCUU5’**

**| | ||.||.||.||**

**5’GGAGGAGAGGAGGAGGAGGAGGUGGUGGUGGAGGAGGAGGCAGGGGGTGGAGAGAGAGAA3’**

**EXT2 (three splicing forms)**

**Sequence alignment between TRIM28-2(Sigma) and three splicing forms of EXT2, NM_000401.3, NM_001178083.1 and NM_207122.1**

**10**

**TRIM28-2(Sigma) 3’CUCUGGUUUGGACACGAAU5’**

**||| |||||||**

**5’CCGGGCCUGUCUGUUUGUUCCCUCCAUCGAUGUGCUUAACCAGAACACACUGCGCAUCAA3’**

**TSPAN7 (one isoform)**

**10**

**TRIM28-1(Sigma) 3’CGAGAUGACCCGGUCGGUU5’**

**| |||||||||| 5’CUAGUGAACCUCACCCCGAGGCCCUGCAUGGGCCAGCCCCUCCAUCUGUACUUGGUCC3’**
